# Investigation of Electromechanical Properties on 3-D Printed Piezoelectric Composite Scaffold Structures

**DOI:** 10.3390/ma14205927

**Published:** 2021-10-09

**Authors:** Tutu Sebastian, Miriam Bach, Andreas Geiger, Tony Lusiola, Lucjan Kozielski, Frank Clemens

**Affiliations:** 1 Laboratory for High Performance Ceramics, Empa, Swiss Federal Laboratories for Materials Science and Technology, Überlandstrasse 129, 8600 Dübendorf, Switzerland; tutusebastian@hotmail.com (T.S.); Miriam.Bach@ikfvw.tu-freiberg.de (M.B.); GeigerAndreas@web.de (A.G.); tlusiola@gmail.com (T.L.); 2 Institute of Ceramics, Refractories and Composite Materials, TU Bergakademie Freiberg, Agricolastraße 17, 09596 Freiberg, Germany; 3 Faculty of Science and Technology, University of Silesia, 1A 75 Pułku Piechoty St., 41-500 Chorzów, Poland; lucjan.kozielski@us.edu.pl

**Keywords:** material extrusion-based additive manufacturing (MEX), fused deposition modeling (FDM), fused filament fabrication (FFF), thermoplastic processing, PZT, BaTiO_3_, ferroelectric composites, transducer

## Abstract

Piezoelectric composites with 3-3 connectivity gathered attraction due to their potential application as an acoustic transducer in medical imaging, non-destructive testing, etc. In this contribution, piezoelectric composites were fabricated with a material extrusion-based additive manufacturing process (MEX), also well-known under the names fused deposition modeling (FDM), fused filament fabrication (FFF) or fused deposition ceramics (FDC). Thermoplastic filaments were used to achieve open and offset printed piezoelectric scaffold structures. Both scaffold structures were printed, debinded and sintered successfully using commercial PZT and BaTiO_3_ powder. For the first time, it could be demonstrated, that using the MEX processing method, closed pore ferroelectric structure can be achieved without pore-former additive. After ceramic processing, the PZT scaffold structures were impregnated with epoxy resin to convert them into composites with 3-3 connectivity. A series of composites with varying ceramic content were achieved by changing the infill parameter during the 3D printing process systematically, and their electromechanical properties were investigated using the electromechanical aix PES device. Also, the Figure of merit (FOM) of these composites was calculated to assess the potential of this material as a candidate for transducer applications. A maximum for the FOM at 25 vol.% of PZT could be observed in this study.

## 1. Introduction

Transforming mechanical energy into electrical energy and vice versa is indispensable in the field of the transducer industry. It has been witnessed since the 1950s that piezoelectric materials based on lead zirconate titanate (PZT) and barium titanate (BT) ceramics are the lead candidates to meet business demands owing to their outstanding electromechanical properties. A wide variety of manufacturing techniques in ceramic processing have been employed to obtain the best piezoelectric properties from these materials. Traditionally, these processing routes strived to obtain highly dense ceramics (>95%) to maximize the electrical and mechanical properties. Most functional properties such as piezoelectric, pyroelectric and dielectric tend to exhibit maximum values when the relative density is close to 100% [1]. However, by introducing a secondary passive phase in the form of low dielectric constant material (polymer or porosity), it is found that certain coupled properties such as longitudinal and transverse piezoelectric effects can be tuned independently [2,3]. For example, in the case of a piezoelectric application such as a hydrophone, the figure of merit for the piezoelectric voltage coefficient (g), which is obtained by dividing piezoelectric strain coefficient (d) by the product of permittivity of free space (ε_0_) and relative permittivity (ε_r_), is an important factor. Hence, a low relative permittivity is essential to maximize the figure of merit in this particular case. Research has proved that transforming monolithic ceramics to piezoelectric composites enhances their applicability due to better acoustic impedance matching and lower dielectric permittivity for ultrasonic applications.

Manufacturing a composite material not only implies choosing the component phases with the right properties but also correctly coupling them [2]. The connectivity between the active ceramic and the passive polymer phase determines the final electromechanical properties of the composite where a total of 10 possible connectivity patterns for a 2-phase system were proposed, in which each phase could be continuous in 0, 1, 2 or 3 dimensions [2]. The internationally accepted nomenclature is (0-0), (0-1), (0-2), (0-3), (1-1), (1-2), (1-3), (2-2), (2-3) and (3-3) where the first number in the parenthesis represents the connectivity of piezoelectric active phase and the second number refers to the polymer inactive phase. A schematic of all these connectivities can be found in [4].

Amongst all these possible connectivities, 0-3, 1-3, 2-2 and 3-3 patterns are extensively used in ultrasound imaging, sensors, hydroacoustic devices, energy harvesting, etc., due to their high flexibility, high hydrostatic voltage coefficient, and better acoustic impedance matching [5,6,7,8,9,10]. Contributions on the fabrication of 0-3 and 1-3 composites using fibrous structures were reported previously [11,12]. Other interesting techniques such as injection molding, dice and fill or freeze casting have also been employed to produce 1-3 and 2-2 composites [13,14,15]. However, the fabrication of a 3-3 composite is more complicated, since active ceramic and passive polymer phases have to build up connectivity in all three directions. One of the popular techniques, the coral replamine technique, involves impregnating a cubic coral structure with wax under vacuum followed by dissolving the coral away using an acid. The porous wax negative is filled with PZT. After thermal treatment, the resulting porous PZT structure is further filled with epoxy resin to obtain 3-3 connectivity [2]. Another important technique, burn-out polymer spheres (BURPS) is using pore-forming agents mixed along with the ceramic powder before the shaping process. The polymer spheres are removed during the thermal treatment and the resulting porous structure is further impregnated with a liquid polymer. After curing the polymer matrix, a 3-3 connectivity is obtained [16,17].

To avoid the complexity in the processing of 3-3 composites, a huge interest in the additive manufacturing of piezoelectric ceramics has been observed in the past two decades [18]. Fused deposition modeling (FDM), based on the new ASTM 52900 now named “material extrusion-based additive manufacturing (MEX)”, is a 3D printing process that extrudes a continuous thermoplastic filament layer by layer to form the final shape of the ceramic part [19]. With computer-aided designs, it is possible to generate complicated models and alter the program according to the end device requirements. For plastics such as acrylonitrile butadiene styrene (ABS), polyamide, polycarbonate, polyethylene and polypropylene, the process of MEX printing is well established.

Due to interpenetration of the ceramic and the polymer phase, the connectivity is maximum in 3-3 composites, and high piezoelectric activity at low dielectric permittivity due to better stress transfer can be achieved [20]. The 3-3 composites offer relatively high sensitivity, low acoustic impedance, high compliance for damping, low density, etc. [21]. However, certain parameters need to be taken into consideration for the application of composites as transducers. The ultrasonic beam transmission capability is reliant on the piezoelectric charge coefficient, d_33,_ and the echo receiving sensitivity is dependent on the piezoelectric voltage coefficient, g_33_. Therefore, large values of piezoelectric coefficients such as d_33_ and g_33_ are highly desirable for these composites to determine their usage as pulse-echo transducers. Hence, some researchers use the product d_33_ × g_33_ as a figure of merit (FOM) to verify its applicability [22,23]. However, the g_33_ value is considered slightly more critical, since a large piezoelectric voltage coefficient allows the intensity of ultrasonic beam to be decreased [24]. A small dielectric permittivity is recommended since it increases the g_33_ value, due to the relation g_33_ = d_33_/(ε_0_* ε_r_), where d_33_ is the piezoelectric charge coefficient, ε_0_ is the permittivity of free space and ε_r_ is the relative permittivity. It allows better electrical impedance matching between the transducer and system instrumentation [25].

Here in this contribution, a simple MEX printing is employed for composite fabrication that consists of continuous transportation of a thermoplastic feedstock filament pushed along rollers through a channel to a heated extruder and passed through a nozzle to the substrate, where it is deposited layer by layer [26]. The process allows the flexibility to vary the ceramic volume fraction in the printed structure by changing the width and spacing within the scaffold. Initially, a fully dense monolithic ceramic of PZT and BT were made using uniaxial pressing, and the electromechanical properties were compared with 3D printed samples having scaffold structure and an offset scaffold structure. Finally, a series of 3-3 composite PZT scaffold structures were prepared with different ceramic contents to determine their effect on piezoelectric voltage coefficient and a figure of merit, FOM (d_33_ × g_33_) to analyze the feasibility of its application as a transducer.

## 2. Materials and Methods

In this study, PZT EC65 (Harris corporation, Melbourne, FL, USA) with a mean particle size of 2.77 μm, a specific surface area (SSA) of 1.33 m^2^/g, and a density of 8.01 g/cm^3^ was used. Additionally, BT (Ferro, GmbH) with a mean particle size of 2 μm, a SSA of 2.21 m^2^/g, and a density of 6.10 g/cm^3^ was selected for the investigations.

PZT (49 vol.%) and BT (52 vol.%) feedstocks were prepared using a high-shear mixer (Haake PolyLab Mixer Rheomix 600, Thermo Fisher Scientific, Karlsruhe, Germany). The thermoplastic binder, in this study, consisted of Ethyl vinyl acetate copolymer Elvax 250 (DuPont, Wilmington, DE, USA) and stearic acid (Sigma Aldrich GmbH, Buchs, Switzerland).

After feedstock preparation, the rheology of both materials was tested using a rotational rheometer (MCR 302, Anton Paar, Buchs, Switzerland) with a plate-on-plate configuration and a 0.5 mm gap [27].

The extrusion of filaments with a diameter of 2.85 mm was performed with the Rosand RH7 Flowmaster (Netzsch GmbH, Selb, Germany). The filaments were extruded and rolled up on a spool to feed the 3D filament printer.

**Figure 1:**
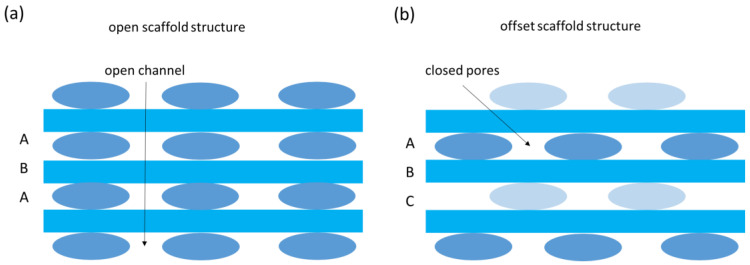
Sketch of 3D printed (**a**) scaffold structure and (**b**) offset scaffold structure.

All 3D printing experiments were performed with a modified Velleman K8200/3Drag kit with a direct extruder Bulldog KL, an E3D hot end and a nozzle size of 0.8 mm. The printing bed consisted of a glass plate, covered with tape (width: 50 mm, length: 50 m, thickness: 0.14 mm; 3D-printerstore.ch). The tape ensures easy removal of the 3D printed scaffold sample. Printing of the scaffold structures was performed at 170 °C with a layer height of 0.25 mm and a printing bed temperature of 60 °C. To achieve better adhesion, the first layer was printed with a layer height of 0.14 mm. Simplify3D was used to generate the scaffold STL-file for the Vellemann printer. Using these parameters for the printing process cylindrical pellets of PZT and BT with a diameter of 20 mm, a thickness of 2 mm a 50% infill, with open and offset scaffold structure could be achieved (Figure 1).

An open scaffold structure (scaffold) consists of an ABA layer organization. Using this configuration, an open, channel-like pore structure between the printed filament can be achieved. This structure can be filled with epoxy material, in a second step. In contrast to this, a closed pore structure (offset) can be achieved using ABC layer configuration. However, it is worthwhile to mention that no pore former needs to be present during the shaping process to achieve a closed pore structure. Additionally, open scaffold structures with different infill percentages, varying from 15% to 70%, were printed and later infiltrated with epoxy resin material.

PZT samples were debinded at 500 °C for 2 h followed by sintering at 1200 °C for 2 h (Pyrotec PY12H, Pyrotec GmbH, Osanbrück, Germany) whereas BT samples were debinded at 500 °C for 2 h and sintered at 1350 °C for 5 h (Nabertherm LHT 04/17, Nabertherm, Lilienthal, Germany). For the debinding process, a heating rate of 3 °C/min until 200 °C followed by 0.25 °C/min until 500 °C was used to allow slow decomposition of thermoplastic binder, present in the samples [28]. To prevent lead volatilization, a lead-rich atmosphere was created inside the sealed crucible by coating it with 0.92 g lead-(II)-zirconate powder (Sigma-Aldrich, Buchs, Switzerland) and 0.08 g zirconia (ITN NANOVATION GmbH, Saarbrücken, Germany), per sample [11]. Furthermore, samples were placed on a PZT powder bed to avoid the sample sticking on to the substrate during sintering. BT was placed on an alumina powder bed for heat treatment, and alumina powder is used to seal off the crucible completely.

Once sintered, PZT scaffolds with different infill (10 to 70 vol.%) were embedded into epoxy Buechler EpoThin™ 2 (ITW Test & Measurement GmbH, Esslingen, Germany) by a casting method, consisting of resin and hardener that are mixed in a volume ratio of 2:1 to fabricate a piezoelectric composite with 3-3 connectivity. For the casting process, the scaffold structures were added to a mould and the liquid epoxy resin was poured inside the mould. The resulted 3-3 composite samples were cured at room temperature inside the mould for at least 9 h. The composite samples were further polished on both sides parallel to each other until first the layer of ceramics was exposed, using the polishing machine (Tegramin 30, Struers GmbH, Birmensdorf, Switzerland) with a 40 µm diamond pad and water.

**Figure 2:**
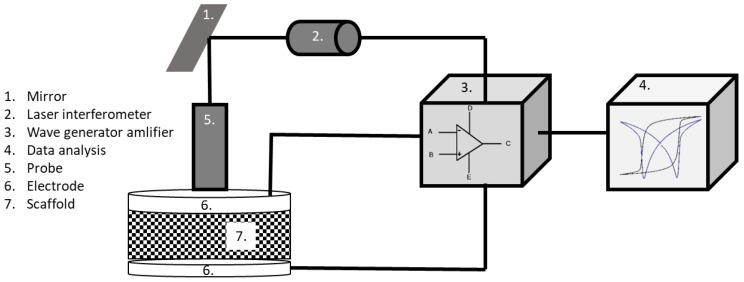
Schematic of the aix PES equipment with a scaffold sample between the electrodes. Low signal analysis (d_33_ and ε) was investigated at low electrical field (<100 V) and 50 Hz. Large signal properties (P-E loop and S-E butterfly curve) were investigated at high electrical fields (up to 2kV) and 0.1 Hz.

Electromechanical characterizations were performed on uniaxial pressed ceramic pellets, scaffold and offset samples. Additionally, PZT epoxy composites with different ceramic content (variation of infill during printing) were investigated by this characterization. All samples were partially electroded with silver paste (Electrodag 5195, Henkel GmbH, Erlinsbach, Switerland) on top and bottom and fired at 150 °C for 15 min to avoid the arcing around the electrodes. The measurements were carried out using the piezoelectric evaluation system aix PES (aixACCT systems GmbH, Aachen, Germany); the details of this setup have been described elsewhere [29,30,31]. A schematic of the instrument is shown in Figure 2. To prevent arcing and extract excessive heat while applying high electric field, samples were immersed in silicone oil (Therm 180, Lauda Dr. R. Wobser GmbH & Co. KG, Lauda-Königshofen, Germany). The large signal polarization and strain hysteresis as a function of the applied electric field was recorded at 0.1 Hz and 1 and 1.5 kV/mm for BT and PZT, respectively, to investigate the effect of the offset scaffold design. For the investigation of the infill content of PZT, the PZT epoxy composite samples were investigated at 0.1 Hz and 2 kV/mm. After these measurements, the composite samples were electrically poled at an electric field of 2kV/mm for 300 s at room temperature. A small-signal stimulus (100 V at 10 Hz) was applied to determine the dielectric constant and the piezoelectric constant, d_33_ and ε_r_, respectively. To evaluate the d_33_, the displacement was measured with a laser interferometer. Furthermore, the cross-section microstructure of the samples was investigated using Scanning Electron Microscopy, (TESCAN SEM Vega3, Tescan GmbH, Dortmund, Germany).

## 3. Results

### 3.1. Rheological Analysis of PZT and BT Feedstock

**Figure 3:**
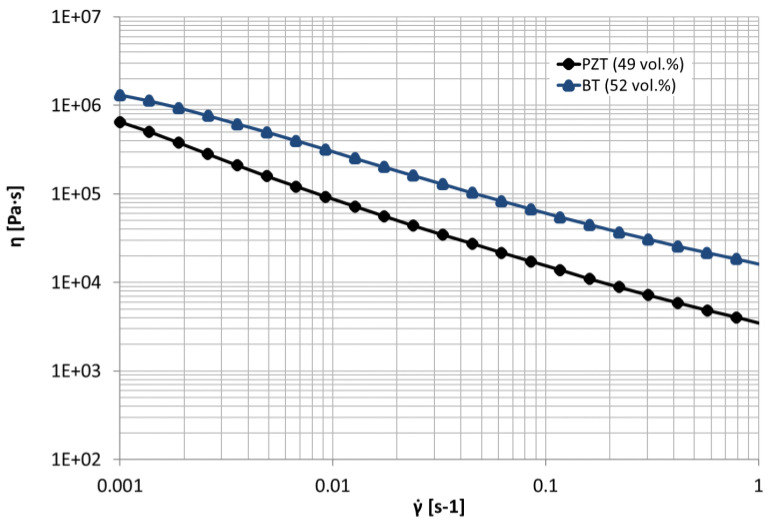
Rheological behavior of two feedstocks, PZT and BT, analyzed with plate-plate rotation rheometer at 150 °C.

After the compounding step, the flow behavior of both feedstocks was investigated by a rotation rheometer (Figure 3).

Both feedstocks show a shear thinning behavior. Due to the similar powder properties, the higher viscosity of the BT feedstock can be explained by the higher solid content. It is worthwhile to mention that a higher PZT content (>49 vol.%) was not able to print with the Vellemann FDM printer.

### 3.2. Comparison of Open and Offset Printed Scaffold Structures with Dense Reference Sample

It is evident that to improve the applicability of piezoelectric material as a transducer, the permittivity of the ceramic material needs to be lowered to improve the piezoelectric voltage coefficient. One of the easiest ways to achieve this is by reducing the ceramic content in the sample or by introducing pores in the structure. In this section, the effect of voids on electromechanical properties of the piezoelectric materials, both PZT and BT, is investigated. For comparison, a fully dense pellet is compared with the two 3D printed scaffolds that have only 50% infill density.

**Figure 4:**
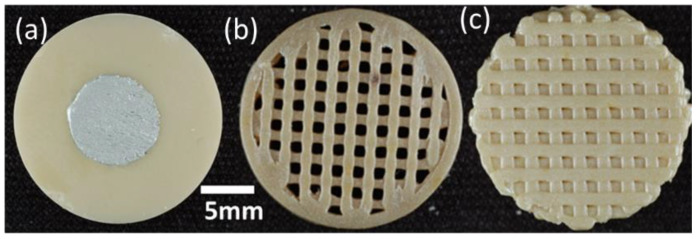
Sintered PZT pellets: (**a**) uniaxially pressed reference sample, (**b**) material extrusion-based (MEX) additive manufactured scaffold structure, and (**c**) 3D printed offset structure.

The 3D printed open scaffold structure (b), where subsequent layers were printed on top of each other, and the offset structure (c), where successive layers are printed with an offset to achieve a structure with closed voids in the end, are shown in Figure 4. 

**Figure 5:**
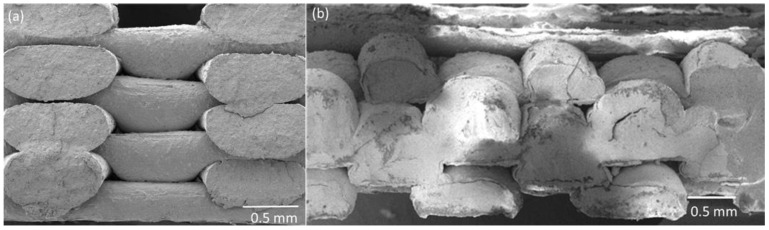
SEM figure of 3D printed (**a**) scaffold structure, and (**b**) 3D printed offset structure.

The open channel structure of the scaffold sample can be easily overserved (Figure 4b), whereas the two other samples do not show an open pore structure. To better show the difference in the layer structure of the two scaffold structures, SEM analyses were performed. Figure 5 shows the SEM pictures of the printed open and offset scaffold structure. As expected, in Figure 5b the offset of the layer A and C can be observed.

**Figure 6:**
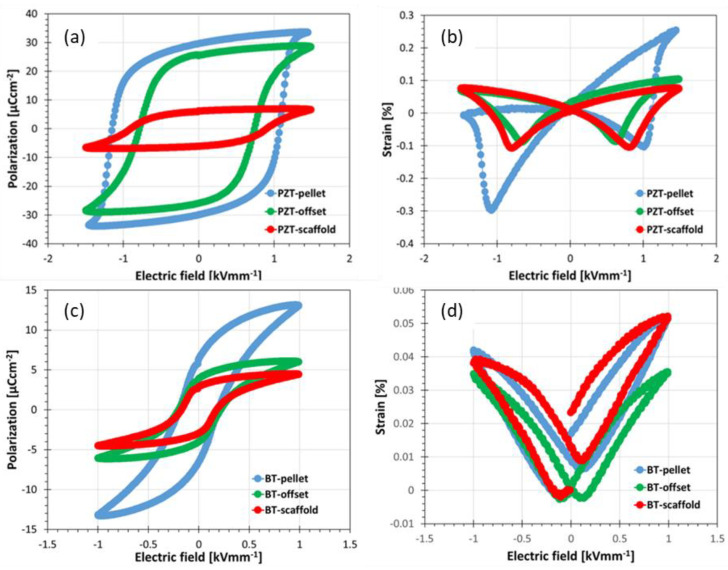
A comparison of piezoelectric properties (**a**) Polarization hysteresis and (**b**) strain hysteresis measured on a pressed sample and 3D printed scaffold structures for PZT; (**c**) polarization hysteresis and (**d**) strain hysteresis measured on a pressed sample and 3D printed scaffold structures for BT.

The electromechanical properties measured on pressed pellets serve as reference values and can be used to compare the effect of voids in the 3D printed samples. In Figure 6, the large field electrical properties (PE and SE loops) of PZT and BT pressed and 3D printed samples are displayed. A considerable decrease in electromechanical properties was observed when comparing the 50 vol.% MEX additive manufactured scaffold and offset sample with the pressed one (pellet) as evident from Table 1. In comparison to the scaffold, the offset printed sample has higher polarization properties. However, no noticeable enhancement in the strain is observed by offset printing.

Generally, in a dense ferroelectric sample, the electric field distribution is more or less homogenous and the dipoles could be aligned correspondingly [32]. However, by introducing low permittivity pores into the sample, the applied electric field tends to concentrate around the pores and thereby an inhomogenous electrical field distribution throughout the structure occurs [33]. It has been reported that the electric fields are inhomogeneous at the pore-ceramic interface where the low field regions exist at the boundaries aligned parallel to the applied electric field vector and high field regions exist at the boundaries aligned perpendicular to the applied electric field vector [34,35,36]. Due to this field inhomogeneity, a reduction in remnant polarization P_r_ and tilting of the polarization-field loops is observed in the MEX additive manufactured 3D printed scaffold and offset samples.

While printing offset grid structures, the voids are covered and form closed pores during sintering. It is well known that porosity in ferroelectric ceramics lowers the breakdown voltage. Therefore, to avoid breakdown of the offset printed structure, a maximal electrical field of 1.5 and 1 kV/mm was employed for PZT and BT, respectively. Due to the volume effect in ferroelectric composites, the decrease of the electromechanical properties of the 3D printed structure that consisted of 50 vol.% ferroelectric phase was expected. Table 1 shows the summary of the piezoelectric properties of the pressed (pellet), the 3D printed open (scaffold) and offset samples.

While printing offset grid structures, the voids are covered with the next layer and form a closed pore scaffold structure. Applying a high electrical field, the air in the pores is polarized and will contribute to the improvement of the polarization properties. In the scaffold structure, the porous channels, filled with silicone oil, can act as a channel for the current to pass through, whereas in the offset printed structure, the current has to pass through the ceramic phase with high dielectric constant and allow the dipoles to organize themselves under the electric field between the electrodes. Finally, looking on the FOM (d_33_ × g_33_) it can be observed that the highest value can be achieved using an open scaffold structure.

### 3.3. PZT-Epoxy Resin Scaffold Structures

**Figure 7:**
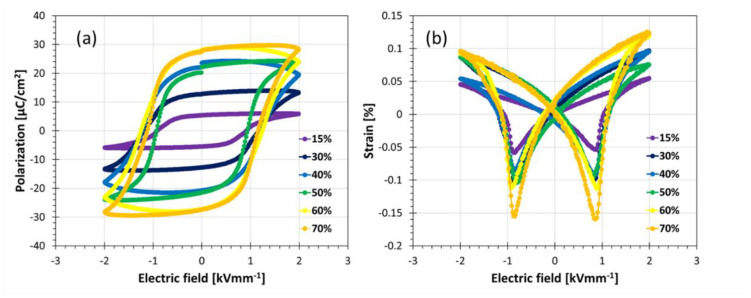
Piezoelectric properties (**a**) polarization hysteresis and (**b**) strain hysteresis of PZT composites with 3-3 connectivity.

Sintered PZT scaffold structures were infiltrated with epoxy resin. A series of composite samples with different ceramic content were investigated, the ferroelectric phase in the composites ranging from 15 to 70 vol.%. Large field electrical properties (PE and SE loops) of PZT composites with 3-3 connectivity are shown in Figure 7.

**Figure 8:**
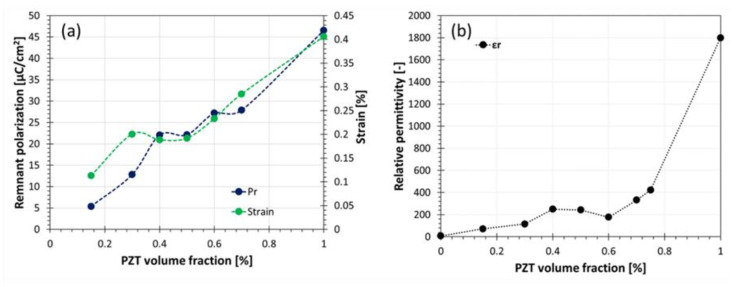
Electromechanical properties (**a**) remnant polarization and maximal strain elongation (**b**) relative permittivity for PZT epoxy piezoelectric composites with 3-3 connectivity as a function of PZT volume content.

As expected, an increase in PZT content inarguably increases the electromechanical and dielectric properties of the composites, as shown in Figure 8. The results are in good agreement with the literature [20].

**Figure 9:**
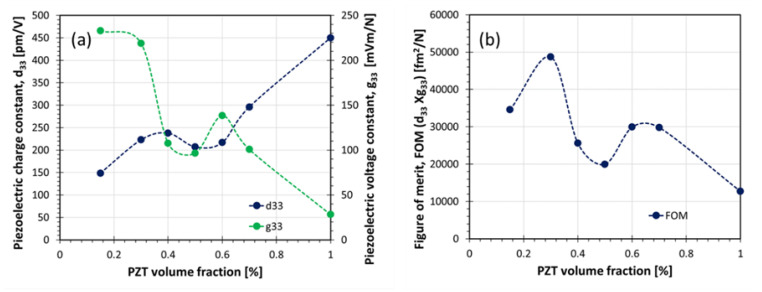
Electromechanical properties (**a**) piezoelectric coefficients, d_33_ and g_33_ and (**b**) FOM, d_33_ × g_33_ for PZT epoxy piezoelectric composites with 3-3 connectivity as a function of PZT volume content.

The analysis of the piezoelectric coefficients d_33_ and g_33_ is shown in Figure 9a. As expected, the g_33_ decreases with higher content of ferroelectric phase, whereas d_33_ increases with higher content of PZT. A considerable increase in g_33_ is observed up to 25 vol.% PZT. This behavior can be attributed to the low dielectric permittivity of the composites in this region. Skinner et al. reported for a 3-3 composite based on PZT and silicone rubber (37 vol.% coral template) a g_33_ of 0.3 Vm/N [3]. In another study, a PZT-vinyl ester resin 3-3 composite, with 18 vol.% template content, achieved a g_33_ of about 0.13 Vm/N by the sacrificial template method [37]. For a PZT-silicone rubber, with 48 vol.% ferroelectric phase, prepared by porous interconnected method, a g_33_ of 0.13 Vm/N was reported [38]. A lead-free 3-3 composite made from (Li,Na,K)NbO_3_/KNbO_3_ composites with Kynol^TM^ using a “two-stage firing” method showed a g_33_ of 0.063 Vm/N [39]. The results presented here are not easy to compare to the literature since the content of the ferroelectric phase inside those composites is not very well reported. Sometimes only the volume fractions of the template are mentioned. In the replica process method, the template is coated with a ceramic slurry and this results typically in a hollow ceramic thread after sintering. Therefore, volume content of the template and ceramic content in the composite are not equal, and a third phase (air voids) has to be taken into account, additionally. However, comparing the result of this study with the reported value by Skinner et al., a similar g_33_ (0.23 Vm/N) could be achieved [3]. Similar g_33_ was obtained by Nagata et al. [38] in their PZT-vinyl ester resin 3-3 composite fabricated by the sacrificial template method. Based on these comparisons, it is feasible to propose that the material extrusion-based additive manufacturing process (MEX) is a very efficient method for the fabrication of transducers.

The figure of merit (d_33_ × g_33_) is presented in Figure 9b. It is noticed that the FOM generally decreased by the increase of the volume fraction of the ferroelectric ceramic. A peak value of about 50,000 fm^2^/N was obtained for the composite with 25 vol.% of ceramic, which could be a potential material for the application as a pulse-echo transducer.

The results, shown in Figure 9b, are in good agreement with the literature [40]. As proposed by Stuber et al., the highest value was observed at low ceramic volume concentration. They proposed a maximum below 10 vol.% for 1-3 composites using the van den Ende model [41] and the Bowen model [42]. It is worthwhile to mention that using the Yamada model [43] for 0-3 composites, the value for d_33_ × g_33_ increases up to 50 vol.% of ferroelectric phase [40]. It can be assumed that the peak at higher filler content in the present study can be explained by the fact that a 3-3 composite structure was used here, whereas the models of van den Ende and Bowen are based on 1-3 a composite structure. In the z-direction, (thickness of the sample) areas consist of only ceramic phase (crossover between 0 and 90 °C printed layers A and B). On the other hand, a laminate structure (2-2 composite) is expected between the crossover points of the printed layers. This could explain the second maxima in the FOM at higher filler content (Figure 9b).

## 4. Conclusions

Material extrusion-based additive manufacturing (MEX) can be used to fabricate transducer composites based on ferroelectric materials for future studies. In comparison to former studies where either polymer templates or a replica technique was used, or porous ceramic structures were achieved using pore formers or partial sintering process, MEX has the advantage to design different 3-3 or 0-3 composite structure with adjustable volume fraction of the ferroelectric phase. For the PZT and BT scaffold structures, a thermoplastic feedstock needs to produce by mixing ceramic, thermoplastic binder and surfactant and extrude this compound into flexible filament. These flexible filaments can be fed into a commercial FDM/MEX printer.

It is worthwhile to mention that replica ceramic processing will result in a three-phase composite. Inside the ceramic structure, a porous core channel due to the polymer template was reported by Olyaei et al. [37]. Based on the electromechanical results achieved with samples, printed with offset structure and the results, achieved by Skinner et al. [3], after breaking the silicone-infiltrated ceramic coral structure, it can be proposed that printing scaffold structures with hollow filaments can improve the transducer properties. The offset structures show better polarization behavior in comparison to the scaffold ones, due to less interaction of electric field between the electrodes.

PZT-epoxy 3-3 connective composites demonstrated that scaffold structures with a wide range of PZT volume fractions can be tailored by simply changing the infill parameter during the printing process. It is seen that the relative permittivity, polarization and piezoelectric charge coefficient of the composites can be tailored in a wide range. However, the piezoelectric voltage constant, g_33_, shows the inverse behavior since low permittivity composites show higher g_33_ values because g_33_ is the quotient between d_33_ and ε_r_. The g_33_ values obtained in this study are quite similar to the ones seen in the literature; however, by using MEX process they can be tailored over a wide range (15 to 70 vol.% PZT ceramic content). An FOM was calculated for different ceramic volume content to analyze the feasibility of this composite as a transducer material. It was found that an FOM peak was observed at 25 vol.% PZT content. Therefore, this composition seems to be interesting for transducer applications.

## Figures and Tables

**Table 1:** A comparison of electrical properties in pellet sample (pressed), 3D printed open (scaffold) and offset (offset) scaffold structure of PZT and BT.

	PZT (1.5 kV/mm)			BT (1 kV/mm)		
	Pellet	Scaffold	Offset	Pellet	Scaffold	Offset
Piezoelectric constant d_33_ (pm/V)	384	202	273	142	107	127
Permittivity ε_r_	1405	297	1177	3657	906	1372
Strain (%)	0.36	0.19	0.18	0.04	0.04	0.04
Remnant polarization P_r_ (μC/cm^2^)	29.7	6.2	25.5	6.2	2.9	3.5
Piezoelectric voltage constant g_33_ (mVm/N)	31	77	26	4	13	10
FOM (d_33_ × g_33_ (fm^2^/N)	11904	15554	7098	568	1391	1270

## Data Availability

The data presented in this study are available on request from the corresponding author.
